# Research on the application of a cement and soil aggregate for the ecological restoration of vegetation in artificial soil

**DOI:** 10.7717/peerj.14657

**Published:** 2023-01-13

**Authors:** Zhuxin Mao, Qian Li, Yuchao Wang

**Affiliations:** 1 Xi’an Botanical Garden of Shaanxi Province, Institute of Botany of Shaanxi Province, Xi’an, PR China; 2 Shaanxi Engineering Research Centre for Conservation and Utilization of Botanical Resources, Xi’an, PR China

**Keywords:** Cutting slops, Vegetation restoration, Artificial soil, Cement, Soil aggregate, Soil quality index

## Abstract

The construction of high-speed roads has resulted in large amounts of steep and exposed cut slopes, posing more potential hazards in areas with mountains and hills. Vegetation restoration is an effective and environmentally-friendly way to restore exposed slopes using outside soil spray seeding, though it is difficult to establish a vegetation cover. Spraying artificial soil on high and steep slopes is a challenging task as it is difficult to keep the fluid mixture on sloped surfaces. Because of these challenges, this study applied different combinations of cement and soil aggregates in artificial soil, measuring final soil properties after one growing season. Experimental results showed that there were substantial differences in all basic soil parameters and in the soil quality index after different treatments. In particular, adding 5–10% cement content could improve the adhesion of artificial soil without remarkably reducing soil quality; adding 0.09% of soil aggregate was also beneficial to soil nutrient availability. These findings indicate that the combination of cement and soil aggregates could be applied in artificial soils for the ecological restoration of steep slope vegetation. Adding cement to the soil increased the alkaline levels of the soil, so it is important to reduce artificial soil pH in the future. The application of a cement and soil aggregate should be considered in the field for the ecological restoration of slope vegetation, and the impact of this addition on slope stability and vegetation growth should be explored with further research.

## Introduction

In China, the rapid construction of high-speed roads has resulted in large amounts of steep and exposed cut slopes (Tong, Liu & Yu, 2014), damaging the original topsoil and vegetation environment through soil erosion and degradation, landslides, and rock displacement, as well as a series of ecological environmental issues (Hjort et al., 2022). Cut slopes pose more potential hazards in areas with mountains and hills. Landslides have caused disastrous traffic accidents; in 2007, 24,993 geomorphological accidents occurred in China, of which 15,478 were landslides (Gao & Sang, 2017) and 10% (1,543) of those were highway landslide disasters (Yin et al., 2020). Nearly 1,100 fatalities and $5–10 billion USD worth of damage have been caused by landslide disasters in China annually since 2000 (Hong et al., 2017). Improving the safety of the exposed cut slopes along the expressway is urgently needed.

In most regions, multiple effective approaches have been applied to restore cut slopes, but vegetation restoration is an effective and environmentally-friendly approach for slope restoration (Stokes et al., 2014). This is because plant roots can resist soil erosion, stranded precipitation, slow runoff, and reduce water infiltration (Tong, Liu & Yu, 2014). Plant roots are also crucial to improving the quality of the slope soil (Chau & Chu, 2017; Li et al., 2018) and enhancing soil erosion resistance (Fattet et al., 2011). Increasing soil surface litter could remarkably improve the physical and chemical properties of the soil, microbial community dynamics (Angst et al., 2017), and the soil water holding capacity, but it is difficult to establish vegetation cover on high and steep slopes through natural succession, so some artificial restoration is needed to restore these exposed slopes (Chen et al., 2016). One common method is the ‘outside soil spray seeding’ method, in which protective nets are affixed and surface hardeners (*e.g.*, cement) are sprayed on the bare cut slopes (Huang et al., 2017). The surface hardeners are mixed with different herb, shrub, and tree seeds, which can retain soil surface litter, reduce soil erosion, and increase the rainfall infiltration rate on the slopes. This type of restoration can improve soil quality and enhance the structure stability of the slopes. The ‘outside soil spray seeding’ method has been applied in southwest and northwest China in areas with steep slopes lacking plant growth (Li et al., 2018). The use of appropriate additives to keep the artificial soils on the surface of the steep slopes is still limited. Adding cement may increase the adhesion of the artificial soil mixtures, preventing soil loss induced by rainwater erosion (Bischetti et al., 2021). The current artificial soil mixtures also do not have effective soil aggregates, likely affecting seed germination and growth. A combination of cement and soil aggregate added to the artificial soil is likely the best, as cement improves the shear strength of the soil and reduces soil loss, and soil aggregate enhances the soil quality of artificial soil mixtures (Chen et al., 2013; Yuan et al., 2016).

In recent years, some researchers have started to study ecological slope restoration using vegetation restoration models (Araújo & Costa, 2013; Chau & Chu, 2017; Karim & Mallik, 2008; Lenka et al., 2012; Matesanz & Valladares, 2007; Yang et al., 2016), while others have focused on the potential of woody plants, especially trees, on the reduction of topsoil landslides (Liang et al., 2017; Stokes et al., 2009). However, the impact of different plant combinations and substrates on ecological vegetation restoration is still unknown. Therefore, it important to explore the best kind of artificial soil to use for prompt plant growth on slopes.

Artificial soils used should have normal physical, chemical, and biological properties, which can be assessed using the soil quality index (SQI) with minimum data sets (MDS; Andrews, Karlen & Cambardella, 2004), allowing researchers to select the representative soil indicators and improve the validity of the evaluation data (Doran & Parkin, 1994). Three steps are applied to calculate soil SQI: (Andrews et al., 2003; Brejda et al., 2000): (1) MDS selection, (2) scoring MDS indicators, and (3) calculating integrated SQI values. It is crucial to perform a principal component analysis (PCA) and Pearson’s correlation analysis, selecting the best and representative indicators for MDS. The SQI index can directly and accurately evaluate soil quality (Xu et al., 2009). As illustrated by previous studies, the SQI effectively evaluates the impact of soil quality on crop yield and soil conditions under different land-use patterns (Askari, O’Rourke & Holden, 2015; Paz-Kagan et al., 2014). For example, the index was able to assess the success of an ecological restoration of rangeland soils in a semi-arid environment, and reveal the vegetation restoration and plant productivity of the abandoned croplands, as found in the Mediterranean region (Imaz et al., 2010; Sánchez-Navarro et al., 2015). More work is needed, however, to determine the sensitivity of the SQI needed for assessing soil quality within other soil types (Imaz et al., 2010).

In this study, the SQI was applied in a warm temperature zone to assess the artificial soil quality with different levels of cement and soil aggregates added. Previous studies have indicated that some fundamental parameters (*e.g.*, soil pH, water content, and organic carbon content) could reflect changes in artificial soil quality. Total nitrogen, phosphorus, and potassium are essential elements for cell metabolism and plant growth (Sung et al., 2015). Available nitrogen, phosphorus, and potassium can be used as indexes to evaluate soil fertility (Tian et al., 2021). Therefore, these essential factors were used as indicators of artificial soil quality in this study. Some indexes for evaluating the structural characteristics of artificial soil were also used, such as supply source and soil storage of effective nutrients (Jones et al., 2013). Microbial carbon in soil, which is closely related to soil nutrition quality (Gu et al., 2009) was also used to assess artificial soil nutrition quality (Tu, Ristaino & Hu, 2006).

In the present study, different levels of cement and soil aggregates were added to artificial soils. These soils were then compared to determine which combination type was the best for the ecological restoration of vegetation, had the highest soil nutrition quality, and increased slope stability the most. The objectives of this study were to (1) use the SQI and the physical/chemical characteristics of the soil to evaluate the structural stability and quality of artificial soils with different cement and soil aggregate combinations; (2) determine the key indicators that can promote the development of artificial soils and the ecological restoration of vegetation. The results of this study provide a theoretical and practical reference for vegetation restoration on cut slopes and further research should test these study results on cut slopes.

## Materials & Methods

### Experimental design

To investigate the effects of cement and soil aggregate on seedling survival and growth, an orthogonal experimental design with two factors was adopted in this study. A PO-32.5 cement (Yaobai, Shaanxi, China) was applied in the experiment with cement content tested at 0%, 5%, 10%, 15%, and 20%, according to the methods outlined by Chen et al. (2013). These levels of cement were combined with different levels of soil aggregate (Lvmeng, Fujian, China), according to the manufacture’s recommendations, with 0.00%, 0.03%, 0.06%, and 0.09% soil aggregate tested. This study used a 5 × 4 orthogonal experiment design; there were 20 groups of treatments with three replicates per treatment for a total of 60 pots in this experiment.

*Elymus nutans*, a perennial herb (Mao et al., 2019), is an ideal pioneer plant for ecological restoration in alpine grassland areas of China (Gu et al., 2009; Tan et al., 2020). Mature *Elymus nutans* seeds were collected from the Qinghai-Tibet Plateau in China (36°57′N, 105°51′E, 3140 m asl) in 2020. There were 60 seeds per pot (pot size: 32.5 cm depth and 25.5 cm diameter), sown by hand to a depth of two cm. The 32.5 cm pot depth was suitable for root growth because the root systems of herbaceous plants are densely distributed in the top 30 cm soil layer (Burylo et al., 2012). The substrate used in this study was a combination of loose, loamy soil (Table 1) and organic matter (3:1). Each pot was filled with a different combination of cement and soil aggregate with the same weight of mixed substrate added, according to the experiment design. The pots were each watered to saturation and then maintained at 60% of the maximum field water holding capacity by weighing the pots daily and replenishing the water to the desired weight. All cells under the experimental treatment were isolated from each other to prevent the competition effect. To minimize the effect of the pot position, the pots were randomly rotated every three days.

**Figure 1:**
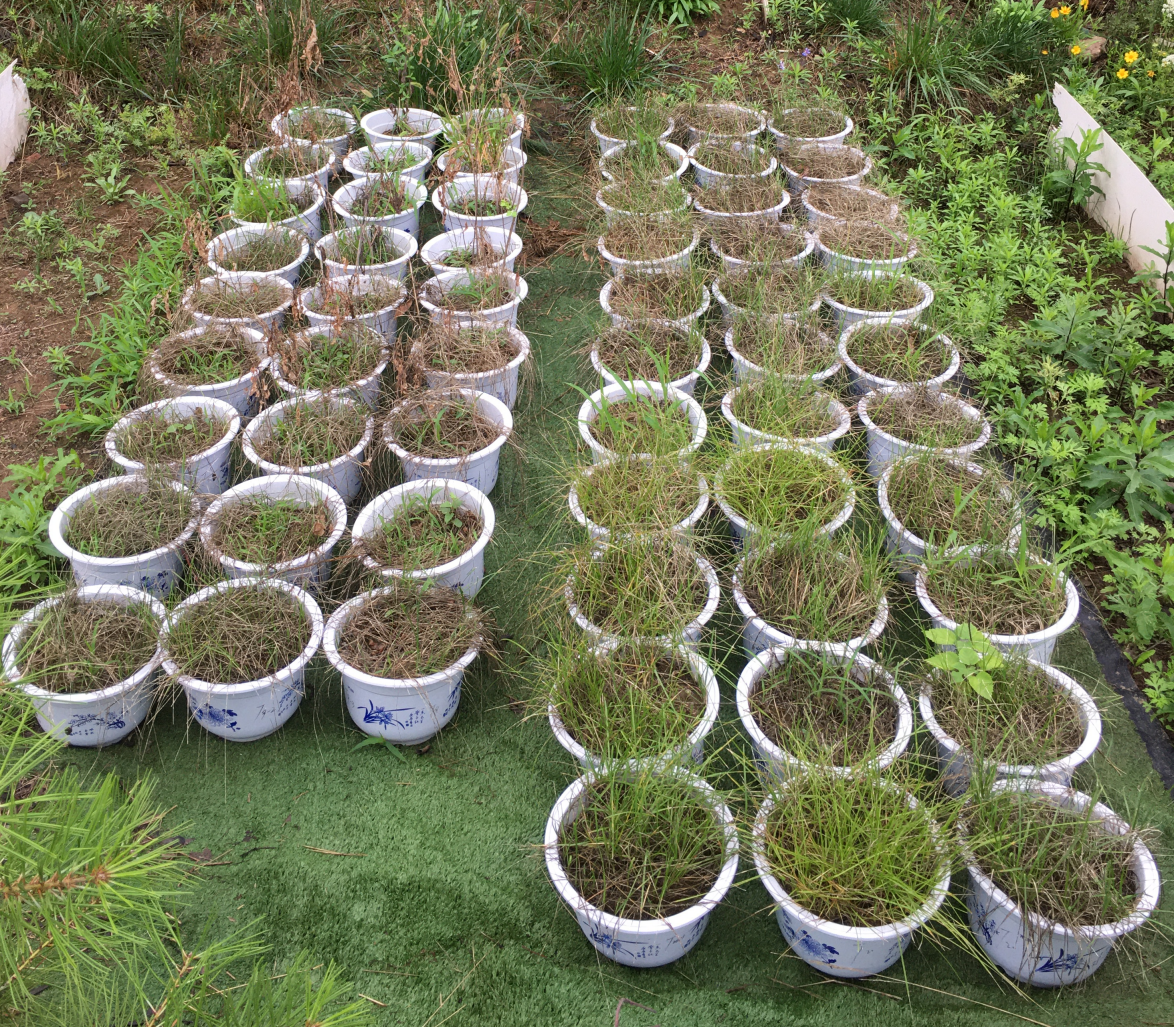
Site and plant growth conditions of the experiment.

After six months of growth (Fig. 1), the plant samples with a stubble height of two cm were cutting by hand during a vigorous growth period. The soil samples were collected, ground, and mixed well; 20% of the soil samples were tested for soil indexes. The dry plant biomass of the plant samples was measured after oven drying (65 °C, 24 h). After removing stones and plant and animal debris, the air-dried soil samples were ground and then passed through a 100 mesh (0.15 mm) nylon screen (Chen et al., 2015). The samples were then stored in a dark place at a low temperature for chemical analysis.

**Table 1:** Physical and chemical traits of loose loamy soil in the experiment.

pH	Organic matter (g/kg)	Total nitrogen (g/kg)	Total phosphorus (g/kg)	Total potassium (g/kg)	Alkali-hydrolyzed nitrogen (mg/kg)	Available phosphorus (mg/kg)	Available potassium (mg/kg)
7.78	18.20	1.69	0.68	20.53	58.20	3.08	165.94

### Sample analysis and experimental methods

The treated samples were preserved and analyzed for the physical-chemical index analyses. The soil organic carbon (SOC) was determined using the potassium dichromate oxidation method. Soil/water suspension (1:2.5 ratio) pH was tested using the potentiometric method. Matric suction and unconfined compressive strength were tested using a densiometric (2100F) and strain-controlled triaxial (TSZ-3) testing apparatus (Zhang et al., 2022). Alkali-hydrolyzed nitrogen (AN) was tested using the alkali solution diffusion method; available phosphorus (AP) was measured using the spectrophotometric colorimetry method (Olsen, 1954); available potassium (AK) was measured using flame Xphotometry; microwave digestion and the Kjeldahl method were used to measure total nitrogen (TN); the sodium hydroxide melt-molybdenum antimony colorimetric method was used to measure total phosphorus (TP); total potassium (TK) was measured using the sodium hydroxide melt-flame photometry method (Zheng et al., 2020); and the concentration of microbial biomass carbon (MBC) was tested using the chloroform fumigation-extraction method (Vance, Brookes & Jenkinson, 1987). Dry soil samples had distilled water added for the microbial activity culture (Wollum, 1983). Soil samples were then divided into fumigated and unfumigated groups with the pretreatment process based on previously published methods (Khan et al., 2010). The microbial biomass carbon (MBC) content was measured using an automated Total Organic Carbon analyzer (Shimazu, TOC-5000, Japan). MBC = 2.22 (C_fumigated_ −C_non fumigated_), in which C was extracted from the fumigated and non-fumigated soil samples, respectively (Khan et al., 2010).

### Evaluation of soil quality indexes

The SQI can assess soil nutrition quality using minimum data sets (MDS; Andrews et al., 2003), which allows investigators to select representative soil indicators to improve the validity of the evaluation data (Doran & Parkin, 1994). Calculating the SQI requires three steps (Andrews et al., 2003; Brejda et al., 2000): (1) selecting the minimum data set (MDS), (2) scoring the MDS indicators, and (3) calculating the integrated SQI values. A principal component analysis (PCA) and a Pearson’s correlation analysis are used to identify the appropriate indicators for selecting the MDS (Doran & Parkin, 1994). According to previous studies (Andrews et al., 2003; Andrews, Karlen & Cambardella, 2004), only principal components (PCs) with eigenvalues ≥1 that explain at least 5% of the total variation can be considered for the MDS (Andrews et al., 2003). Within each PC, the indicators with absolute loading values in the highest 10% (Bastida et al., 2006) or indicators that were well correlated (*r* ≥ 0.6) were selected as vital indicators (Andrews, Karlen & Cambardella, 2004). After selecting the indicators of the MDS, a non-linear scoring function was used to transform the soil indicators into scores that ranged from 0 to 1. The sigmoidal function (Eq. (1)) was used as follows (Andrews, Karlen & Cambardella, 2004):

In this study, it was assumed that different soil parameters played various roles in maintaining soil quality, so the soil quality of different treatments was determined using the SQI, which was calculated using the selected soil factor membership values and their weights, as presented below (Andrews et al., 2003; Lin et al., 2017; Masto et al., 2008):

(1) \begin{eqnarray*}SQI=\sum _{i=1}^{n}{W}_{i}\times Q({x}_{i})\end{eqnarray*}

where *W*_*i*_ denotes the weight of soil quality factor *i* (soil property), *Q* (*x*_*i*_) represents the membership value of each soil quality factor, and *n* signifies the number of the selected soil quality factors. The *Q* (*x*_*i*_) values were assessed with different variation functions (Zheng et al., 2005). The pH value was a decreasing function, where the higher the pH value, the higher the alkalization degree; an ascending function was applied for SOC, AN, AP, AK, TN, TP, TK, and MBC (Xu et al., 2009), where higher values indicated a greater contribution to the SQI. The ascending and descending functions were calculated using the following formulas:

(2) \begin{eqnarray*}& & Q({x}_{i})=({x}_{ij}-{x}_{i\text{min}})/({x}_{i\text{max}}-{x}_{i\text{min}})\end{eqnarray*}

(3) \begin{eqnarray*}& & Q({x}_{1})=({x}_{i\text{max}}-{x}_{ij})/({x}_{i\text{max}}-{x}_{i\text{min}})\end{eqnarray*}

where *x*_*ij*_ denotes the value of the selected fundamental parameters for the SQI calculation; *x*_*imax*_ and *x*_*imin*_ represents the maximum and minimum values of the soil property *i* under various combinations of cement and soil aggregate.

In this study, a PCA analysis was employed to determine the component capacity score coefficient, and the weight of the soil quality factor (*W*_*i*_) was calculated according to the score coefficient, as presented below (Xu et al., 2009):

(4) \begin{eqnarray*}{W}_{i}= \frac{{C}_{i}}{\sum _{i=1}^{n}({C}_{i})} \end{eqnarray*}

where *C*_*i*_ represents the score coefficient of soil quality factor *i* while n denotes the number of selected soil quality factors.

### Data analysis

Analysis of variance was used to compare the soil properties under various treatments. Fisher’s least substantial difference (LSD) was used for the mean separation at 0.05 or 0.01, Spearman’s test was used to determine correlations among soil properties, and linear regression was used to calculate the PSD fractal dimensions. All analyses were conducted using the SPSS software (19.0; SPSS, Inc., Chicago, IL, USA).

The statistical analysis was conducted using SPSS Ver. 19. An analysis of variance was applied to compare the soil properties under different treatments, Fisher’s least substantial difference was used for the mean separation at a significance level of *P* < 0.05 or *P* < 0.01, a linear regression analysis was used to measure the physical and chemical soil properties, a Pearson’s test determined the correlations between selected soil parameters, and a PCA analysis was used for factor extraction. Three replicates were separated for independent testing in the laboratory and for statistical analysis.

## Results

### Biomass and soil weight

**Figure 2:**
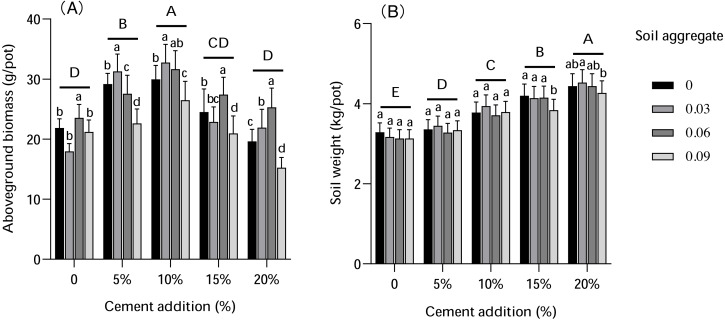
The influence of cement and soil aggregate addition on aboveground biomass and soil weight per pot. (A) Aboveground biomass. (B) Soil weight per pot. Different uppercase letters indicate statistically significant difference between cement additions (*P* < 0.01), different lowercase letters indicate statistically significant difference between soil aggregate treatments (*P* < 0.01).

The results indicated that cement had a more dramatic impact on biomass and soil weight than the control (CK), as shown in Fig. 2. Increasing cement content levels in the soil first increased the biomass of the pots, but then the biomass descended gradually. The maximum biomass (26.50–32.77 g DW/pot) was obtained under 10% cement treatment, which was a statistically higher biomass (*P* < 0.01) compared with other cement percentages. Soil weight also increased as cement percentage increased, with soil weight differing significantly (*P* < 0.01) under different cement percentages. The analysis of variance further indicated that the addition of cement significantly impacted (*P* < 0.01) both biomass and soil weight.

The soil aggregate additions had a varied impact on biomass and soil weight. With the same percentage of cement added, the influence of soil aggregates on biomass was more substantial at low concentrations (0.03% and 0.06%); the biomass after 0.09% soil aggregate addition was lower than CK. In contrast, soil weight was higher with the addition of lower concentrations of soil aggregate compared to CK, but the difference was not statistically significant.

### Soil physical properties

**Figure 3:**
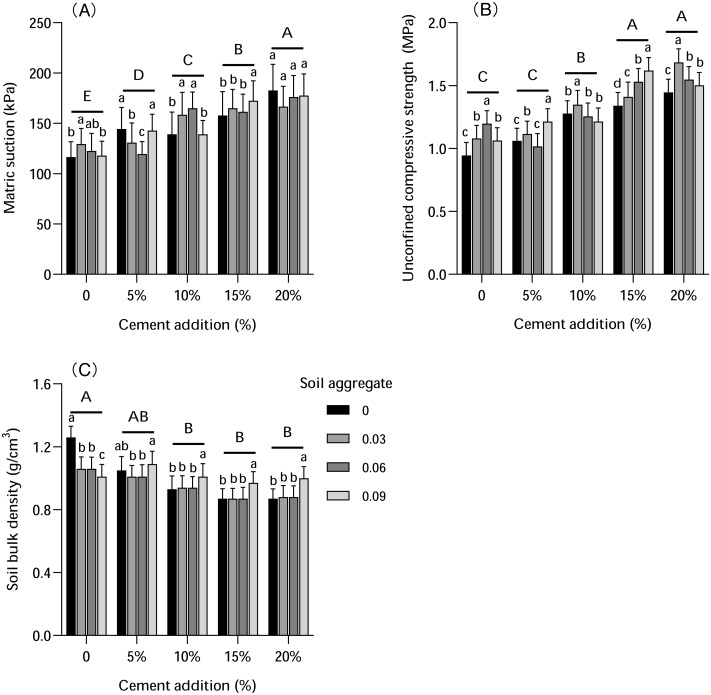
The influence of cement and soil aggregate addition on soil physical properties. (A) Matric suction. (B) Unconfined compressive strength. (C) Soil bulk densAity. Different uppercase letters indicate statistically significant difference between cement additions (*P* < 0.01), different lowercase letters indicate statistically significant difference between soil aggregate treatments (*P* < 0.01).

Soil physical properties were influenced by both cement and soil aggregate addition (Fig. 3). Matric suction gradually increased with cement addition, and there were significant differences between different levels of added cement (*P* < 0.01). The highest matric suction was observed with 20% cement addition, which was 29%–57% higher than CK. The same trend was found in unconfined compressive strength, which increased with cement addition. There was no significant difference between 5% cement addition and CK, but unconfined compressive strength was significantly higher with a 10% cement addition than with a 5% cement addition and significantly lower than a 15%–20% cement addition, and there was no significant difference between 15% and 20% cement addition. Conversely, the level of soil bulk density decreased with cement addition. The soil bulk density level after a 10%–20% cement addition was significantly lower than in CK, but there was no significant difference in soil bulk density between 10% and 20% cement additions.

The influence of soil aggregate on different soil physical properties varied (Fig. 3). Without cement addition, the addition of soil aggregate made the matric suction stronger than in CK. Matric suction value was higher in the 0.03% soil aggregate pot than in both the 0.06% and 0.09%. However, with more cement addition, soil aggregate addition made the matric suction stronger than in both the 10% and 15% cement addition pots, but weaker than the 5% and 20% cement addition pots. The analysis further revealed that statistical significance varied among the treatments. The influence of soil aggregate on unconfined compressive strength was more consistent: unconfined strength increased with more cement addition. Soil bulk density was less influenced by soil aggregates: without cement addition, soil bulk density decreased significantly; with more cement addition, the soil bulk density value was relatively stable, but increased significantly with 0.09% soil aggregate (3.75%–14.22% higher than in CK).

### Soil Ph, SOC, and BMC

**Figure 4:**
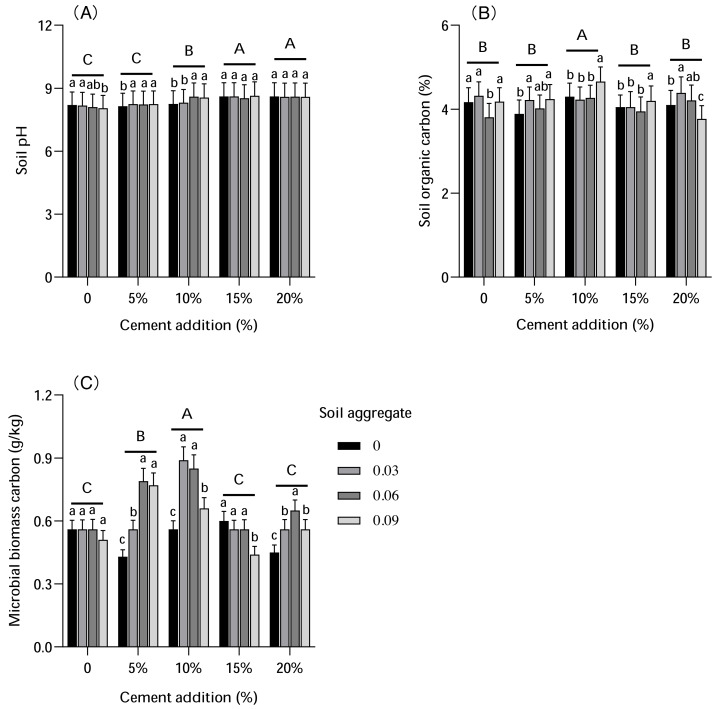
The influence of cement and soil aggregate addition on soil pH, soil organic carbon and microbial biomass carbon. (A) Soil pH. (B) Soil organic carbon. (C) Microbial biomass carbon. Different uppercase letters indicate statistically significant difference between cement additions (*P* < 0.01), different lowercase letters indicate statistically significant difference between soil aggregate treatments (*P* < 0.01).

The influence of cement and soil aggregate on soil pH, SOC, and BMC is shown in Fig. 4. These results revealed that soil pH increased with cement addition and varied between 8.05 and 8.64. Both 5% and 10% cement addition had less influence on soil Ph; 15% and 20% cement addition increased Ph levels, but not significantly. In contrast, the SOC content with 10% cement addition was higher compared with other treatments, whereas the impact of soil aggregate on the SOC content was more significant. The SOC content with 0.09% soil aggregate addition under 0–15% cement was significantly higher compared with other treatments.

The impacts of various soil aggregate treatments on MBC were not significantly different, but the MBC content was higher under 5% (0.56–0.79 mg/kg) and 10% (0.66–0.89 mg/kg) cement addition treatments compared with CK (0.51–0.56 mg/kg). The results indicate that cement and soil aggregates might have an interaction effect on MBC, which was remarkable with a low cement addition and higher soil aggregate addition.

### Plant element

**Figure 5:**
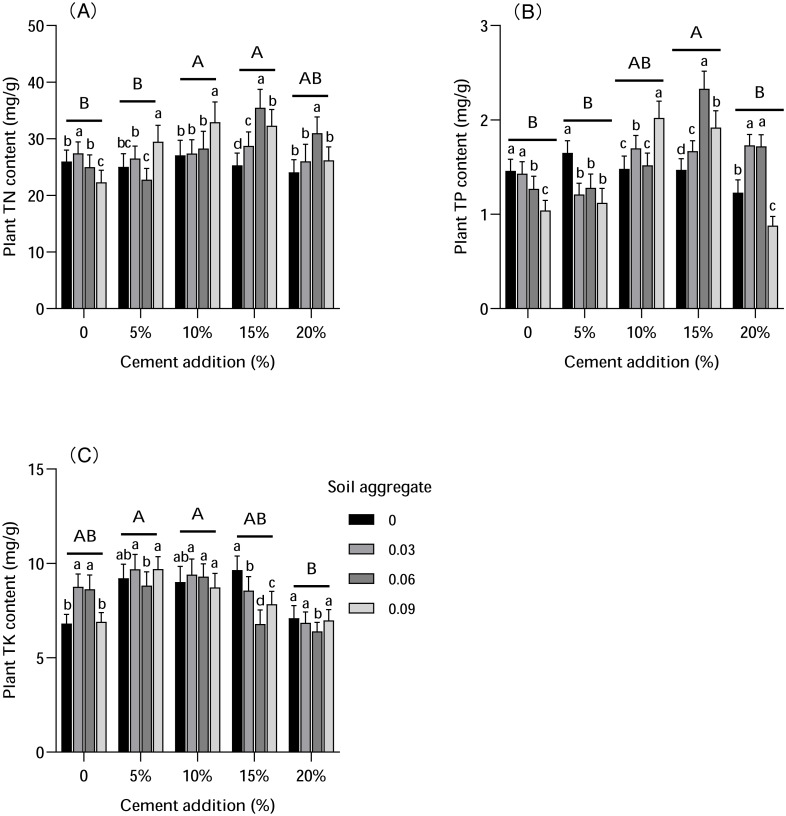
The influence of cement and soil aggregate addition on plant elements. (A) TN. (B) TP. (C) TK. Different uppercase letters indicate statistically significant difference between cement additions (*P* < 0.01), different lowercase letters indicate statistically significant difference between soil aggregate treatments (*P* < 0.01). TN, total nitrogen; TP, total phosphorus; TK, total potassium.

Cement and soil aggregate addition had different influences on plant elements (N, P, and K, as presented in Fig. 5), with the most significant differences found in N and P. Specifically, N content increased with increased cement and soil aggregate content, with N content reaching the highest level with 10%–20% cement addition and 0.06% soil aggregate addition. P content increased with cement addition, but decreased with soil aggregate addition in CK, with impacts to P content varying under other treatments. Similarly, K content was influenced by both cement and soil aggregates, increasing with 5% and 10% cement addition but decreasing with 15% and 20% cement addition. K content in CK increased with 5% cement addition, maintained with 10% cement addition, and decreased with 15% and 20% cement addition.

### Soil element

**Figure 6:**
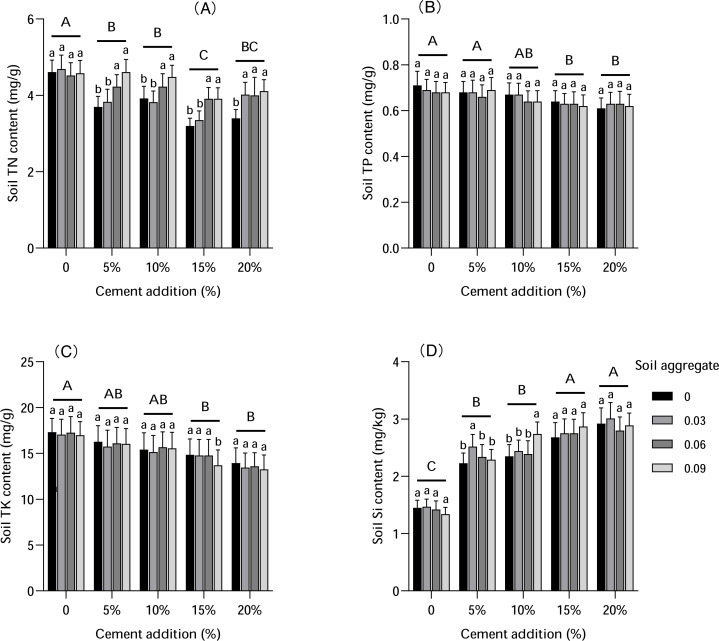
The influence of cement and soil aggregate addition on soil elements. (A) TN. (B) TP. (C) TK. (D) Si. Different uppercase letters indicate statistically significant difference between cement additions (*P* < 0.01), different lowercase letters indicate statistically significant difference between soil aggregate treatments (*P* < 0.01). TN, total nitrogen; TP, total phosphorus; TK, total potassium.

Soil element contents were influenced by both cement and soil aggregate addition (Fig. 6). The N level decreased with increased cement addition, with N levels significantly lower with 15%–20% cement addition compared with CK. Soil aggregates had no substantial influence on N levels without cement addition, but this interaction of cement and soil aggregate varied under different cement additions. The N level increased with increased soil aggregate content, especially with low cement addition. The influence of cement and soil aggregates on P level was roughly consistent. P level decreased with the addition of cement and plummeted with 15%–20% cement addition, but was slightly affected by different soil aggregate additions.

K level trends were more consistent. The K level decreased significantly with increased cement, but there were no changes to K levels observed under different soil aggregate additions, indicating that cement had more influence on K content than soil aggregates. Total soil silicon (Si) content increased significantly with cement addition (*P* < 0.01), but did not significantly differ under different soil aggregate additions.

### Available soil nutrients

**Figure 7:**
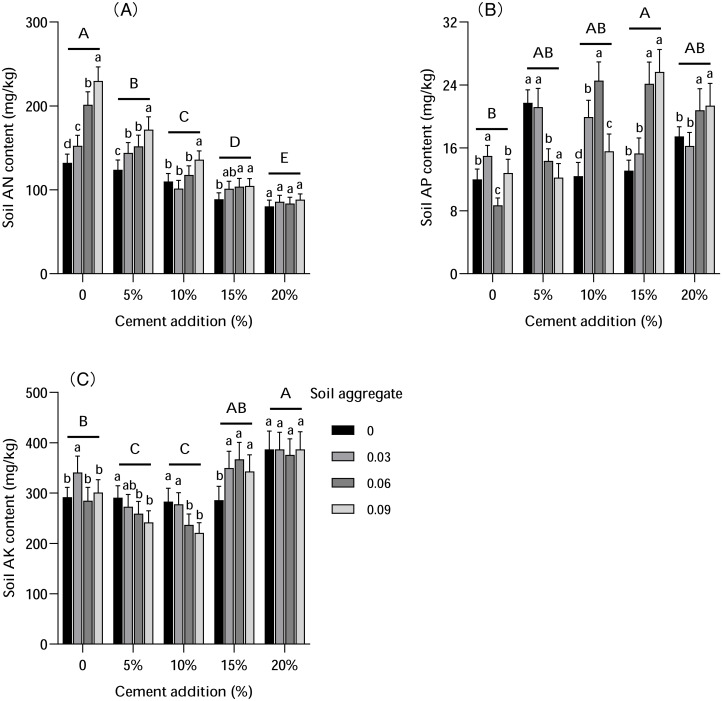
The influence of cement and soil aggregate addition on soil available nutrients. (A) AN. (B) AP. (C) AK. Different uppercase letters indicate statistically significant difference between cement additions (*P* < 0.01), different lowercase letters indicate statistically significant difference between soil aggregate treatments (*P* < 0.01). AN, alkali-hydrolyzed nitrogen; AP, available phosphorus; AK, available potassium.

Cement and soil aggregates both impacted available nutrients in the soil (Fig. 7). AN content decreased with cement addition (5%–20%), with decreases significant with low cement levels but not significant with high cement levels. In contrast, the AN level increased with soil aggregate addition, with significant increases observed with less cement addition (5% and 10%), but insignificant increases with higher cement addition (15% and 20%).

Soil AP content increased with cement addition, with varied increases under different treatments. The highest level of AP was found with 5% cement addition (with 0 and 0.03% soil aggregate) and 15% cement addition (with 0.06% and 0.09% soil aggregate). However, soil aggregates had a different influence on AP, which was more significant with less soil aggregate addition and 5% cement addition as well as with moderate soil aggregate addition with 15% cement addition. These results might indicate that there is an interaction effect of cement and soil aggregate addition on AP.

AK was significantly impacted by both cement and soil aggregates. Compared with the control group, the AK content was lower with 5%–10% cement addition but higher with 15%–20% cement addition, being lowest with 10% cement addition and highest with 20% cement addition. With increased soil aggregates, AK level was higher with 5% and 10% cement addition, lower with 15% cement addition, and stable with 20% cement addition. The variance analysis indicated that the AK level with 15% and 20% cement addition was substantially higher (*P* < 0.01) than with 5% and 10% cement addition.

### The influence on the SQI of artificial soils

SQI was introduced to explore the effect of the combination of cement and soil aggregates on artificial soils. The first step was to obtain the constant weight of the soil parameters. After a Varimax rotation, a PCA analysis produced four principal components (PCs; Table 2). PC1-4 explained 80.54% of the variability in the measured soil parameters, most representing the original parameters. Therefore, the weight of each parameter was calculated through PC1-4 in this study (Table 2). After determining the weight of each soil index, the membership value of each soil index [*Q* (*x*_*i*_)] was determined using Eqs. (2) and (3). Finally, Eq. (1) was applied to calculate the SQI (Table 3). The results indicated that SQI value decreased with cement addition but increased with moderate soil aggregate addition, with the highest value in CK (0.53–0.58) and the lowest with 20% cement addition (0.19–0.34).

**Table 2:** Results of principal component analysis of soil quality indicators.

**Principal components**	**PC1**	**PC2**	**PC3**	**PC4**	Score coefficient	Weight
**Eigenvalue**	4.209	1.335	1.291	1.219		
**Percent**	42.09%	13.35%	12.91%	12.19%		
**Eigen vectors**	**Loading values**					
pH	0.4179	0.0817	0.0665	0.2726	0.2838	11.24%
SOC	0.0315	0.7933	0.0696	0.0467	0.1662	6.58%
MBC	0.0528	0.0397	0.8130	0.1968	0.1943	7.69%
TN	0.1950	0.4205	0.1152	0.5040	0.2664	10.55%
TP	0.3492	0.3584	0.0335	0.2219	0.2809	11.12%
TK	0.4617	0.0617	0.0647	0.0968	0.2765	10.95%
AN	0.4046	0.0228	0.0655	0.1738	0.2520	9.98%
AP	0.1974	0.0753	0.1105	0.6205	0.2273	9.00%
AK	0.2863	0.2135	0.5425	0.2722	0.3132	12.40%
Si	0.4047	0.0388	0.0198	0.2877	0.2647	10.48%

**Notes.**

The high loading values (absolute values) represent the dominant factor in the soil. PC1, first principal component; PC2, second principal component; PC3, third principal component; PC4, fourth principal component.

SOC soil organic carbon MBC microbial biomass carbon TN total nitrogen TP total phosphorus TK total potassium AN available nitrogen AP available phosphorus AK available potassium

**Table 3:** Soil quality index of artificial soil with cement and soil aggregate addition.

Soil aggregate Cement	0%	0.03%	0.06%	0.09%
0	0.53	0.58	0.57	0.58
5%	0.40	0.36	0.41	0.47
10%	0.36	0.40	0.37	0.39
15%	0.24	0.27	0.30	0.26
20%	0.19	0.27	0.34	0.27

To further distinguish the relationship among the different soil physicochemical properties, Pearson’s correlation coefficients were applied in the analysis (Table 4). The results showed that Si content was positively correlated with pH (*P* < 0.01), AP, and AK, but negatively correlated with TN, TP, TK, and AN. Conversely, TN was positively correlated with TP, TK, and AN; TP was positively correlated with TK and AN; and TK was positively correlated with AN. In summary, TN, TP, TK, and AN were positively correlated with each other, but negatively correlated with Si. Cement addition, the primary source of Si and an accelerant for soil solidification, restricts TN, TP, TK, and AN levels, explaining the relationship between Si and other soil physiochemical properties. In addition, soil pH was negatively correlated with the TN, TP, TK, and AN of artificial soil (Table 4), while TN content was positively correlated with TP, TK, and AN. Soil pH was also positively correlated with SMC, which was related to cement addition, as shown in Table 4. In conclusion, the Pearson correlation indicated that there was a positive correlation between soil physical and chemical parameters.

**Table 4:** Pearson’s correlation coefficients among different soil physicochemical properties.

	Mean	STD	pH	SOC	MBC	TN	TP	TK	AN	AP
pH	8.389	0.213	1							
SOC	4.151	0.289	0.119	1						
MBC	0.619	0.179	0.048	0.125	1					
TN	4.106	0.458	−0.388^**^	0.240	0.214	1				
TP	0.659	0.034	−0.669^**^	0.230	0.044	0.569^**^	1			
TK	15.349	1.304	−0.811^**^	0.008	0.016	0.473^**^	0.735^**^	1		
AN	125.498	41.926	−0.713^**^	−0.002	0.022	0.504^**^	0.517^**^	0.754^**^	1	
AP	17.228	6.036	0.511^**^	0.027	−0.097	−0.320^*^	−0.402^**^	−0.438^**^	−0.445^**^	1
AK	309.322	59.778	0.302^*^	−0.232	−0.352^**^	−0.250	−0.479^**^	−0.544^**^	−0.436^**^	0.200
Si	23.744	5.629	0.780^**^	0.062	−0.005	−0.495^**^	−0.541^**^	−0.841^**^	−0.722^**^	0.464^**^

**Notes.**

* *p* < 0.05

** *p* < 0.01

SOC soil organic carbon MBC microbial biomass carbon TN total nitrogen TP total phosphorus TK total potassium AN available nitrogen AP available phosphorus AK available potassium

## Discussion

Our results illustrated a positive correlation between soil physical and chemical parameters while considering the weight coefficient, suggesting that chemical parameters play a more essential role in improving soil quality than physical properties. Therefore, improving artificial soil quality and promoting vegetation ecology restoration should focus on improving the physical and chemical parameters of the soil. In this study, the physical properties of the soil were influenced by both cement and soil aggregate addition, with levels increasing with increased cement addition. As for chemical properties, cement addition impacted PH, TP, TK, AN, AK, and Si, with different relationships observed between these properties. Further analysis indicated that pH was negatively correlated with the TN, TP, TK, and AN of artificial soil, while TN content was positively correlated with TP, TK, and AN, consistent with previous research (Barre et al., 2017; Wiesmeier et al., 2014). These results could be explained as follows: (1) higher TN content could provide more N resources (Davidson & Janssens, 2006) and increase AN content in artificial soils, thereby promoting plant growth and increasing the input of plant protein; (2) cement addition could raise soil pH and reduce the level of TN, TP, and TK, indicating that more cement is not beneficial to soil nutrients because it decreases soil fertility by limiting N mineralization and release (Caravaca et al., 2017).

SQI was applied to assess artificial soil quality, which was established through a PCA analysis and score function analysis combined with soil information (Xu et al., 2009; Zheng et al., 2005). We found that less cement and more soil aggregate addition resulted in a higher SQI of artificial soils. However, less cement reduced the adhesion of artificial soil mixtures and the structure stability of slopes (Tan et al., 2020). Although proper soil aggregate addition was improved soil nutrition, the influence was largely limited. Thus, more effective management measures should be taken to reduce the effect of cement on the physicochemical characteristics of soil.

In practical application, more cement addition reduces the soil nutrition quality and response to soil solidification, decreasing root respiration and limiting plant growth (Ji et al., 2014). Soil pH was positively correlated with Si. A previous study (Andrews, Karlen & Cambardella, 2004) found that the optimal pH value of soil is between 6 and 7, but the pH levels of all treatments in this study were remarkably higher than that range. In this study, soil pH was an essential parameter of artificial soils, as optimal pH levels help soil optimization, but soil aggregates could not reduce the pH of artificial soils. Sulfur fertilizer effectively reduces soil pH and also provides an essential nutrient for plant growth (Mahar et al., 2016; Sönmez, Turan & Kaya, 2016). Therefore, in future experiments, the addition of sulfur fertilizer to artificial soils should be studied.

For vegetation restoration, it is important to maintain adequate soil nutrition and a healthy root environment, which requires restricting cement addition, but this reduces the stability of slopes and increases soil erosion. Applying biochar and slow-release fertilizer to artificial soil may effectively reduce alkaline soil and improve nutrient use efficiency (Atkinson, Fitzgerald & Hipps, 2010), as well as provide a more stable and long-term supply of carbon, nitrogen, and phosphorus nutrients (Atkinson, Fitzgerald & Hipps, 2010; Yilmaz & Sönmez, 2017). Thus, in future studies of ecological vegetation restoration, the composition of artificial soils should be adjusted appropriately using fertilizers.

## Conclusions

This study aimed to identify the optimal combination of cement and soil aggregate in artificial soils for the ecological restoration of vegetation. The results suggested that different levels of cement addition had different impacts on the nutrition of artificial soils, but a low cement concentration had no significant effect on soil properties. The results comparing the physical and chemical soil properties with the SQI index indicated that herbs could tolerate the addition of 5% and 10% cement. However, TN, TP, TK, and AK are negatively correlated with soil pH, with soils more alkaline with more cement addition, meaning that more cement addition had an adverse effect on soil chemical properties. Furthermore, with cement addition, the SQI index was substantially reduced, but this impact was remedied with soil aggregate addition. This finding supports the previous conclusion that cement + soil aggregates is a better choice for ecological vegetation restoration. The more important finding of this study was that an optimal combination of cement content and soil aggregate could improve the basic parameters and structural properties of the slope soil, preventing landslides and soil erosion. Therefore, the results of this study are a valuable reference for vegetation restoration in warm and humid regions, and the optimal treatment found in this study could be applied in regions with the same climate and soil type as the study areas.

In order to promote the ecological restoration of steep slope vegetation, we make the following recommendations: (1) cement and soil aggregate can be applied in artificial soils for the ecological restoration of vegetation; (2) adding 5%–10% cement could improve the adhesion of artificial soils; (3) and 0.09% soil aggregate addition is beneficial to soil nutrient availability.

This study did not monitor the ecological stability of slope vegetation over a long period of time. Thus, further work needs to study slopes with different gradients to monitor the long-term ecological stability of slope vegetation. Testing the feasibility and outcome of the application of cement and soil aggregate in the field could effectively prompt future development of slope vegetation restoration.

##  Supplemental Information

10.7717/peerj.14657/supp-1 Data S1 Raw Data

## Additional Information and Declarations

### Data Availability

The following information was supplied regarding data availability: The raw data is available in the Supplemental File.
